# Rapid *in situ* imaging and whole genome sequencing of biofilm in neonatal feeding tubes: A clinical proof of concept

**DOI:** 10.1038/s41598-017-15769-9

**Published:** 2017-11-21

**Authors:** Pauline Ogrodzki, Chi Shing Cheung, Mohamed Saad, Khaled Dahmani, Rebecca Coxill, Haida Liang, Stephen j. Forsythe

**Affiliations:** School of Science and Technology, Nottingham Trent University, Clifton Lane, Nottingham, NG11 8NS UK

## Abstract

The bacterial flora of nasogastric feeding tubes and faecal samples were analysed for a low-birth weight (725 g) neonate EGA 25 weeks in intensive care. Samples were collected at age 6 and 8 weeks of life. Optical coherence tomography (OCT) was used to visualise bacterial biofilms inside the nasogastric feeding tubes. The biofilm was heterogeneously distributed along the tube lumen wall, and had a depth of up to 500 µm. The bacterial biofilm and faecal samples included *Enterococcus faecalis* and *Enterobacter hormaechei*. Representative strains, recovered from both feeding tubes and faecal samples, were whole genome sequenced using Illumina, Mi-Seq, which revealed indistinguishable strains, each with less than 28 SNP differences, of *E. faecalis* and *E. hormaechei*. The *E. faecalis* strains were from two sequence types (ST191 and ST211) and encoded for a number of traits related to biofilm formation (*BopD*), adherence (*Epb* pili), virulence (*cps* loci, gelatinase, *SprE*) and antibiotic resistances (IsaA, tetM). The *E. hormaechei* were all ST106, and encoded for *bla*ACT-15 β–lactamase and fosfomycin resistance (*fosA*). This proof of concept study demonstrates that bacterial flora within the neonatal feeding tubes may influence the bacterial colonisation of the intestinal tract and can be visualised non-destructively using OCT.

## Introduction

Bacteria are well known for being able to form biofilms on inert surfaces such as plastic and glass^1,2^, and hence on medical devices including catheters, ventilator tubes, and enteral feeding tubes^3^. Biofilms form when bacteria attach to a surface and then continue to grow forming a layer composed of extracellular materials, such as polysaccharides. Such biofilms are recalcitrant to antibiotics, and are hard to physically remove^4^. Consequently, they can form bacterial loci resulting in chronic and severe infections of patients.

Bacterial biofilm formation on medical devices contributes to hospital-acquired infections (HAI) resulting in prolonged stays in hospital and debilitating infections. HAI such as urinary tract infections due to catheters account for 17% of HAI infections and have a considerable economic expense due to prolonged hospital stays, medical treatment as well as decreased quality of life^5^, severely affecting hospital infection and control measures, including those aimed to reduce antimicrobial resistance.

Premature babies lack a developed immune system and are highly susceptible to infections. Such neonates have increased permeability of their intestinal mucosa, and an undeveloped microflora of the gut^6–9^. This increases the vulnerability of neonates to colonisation and subsequent infections when exposed to opportunistic bacterial pathogens. These infections may not only be localised in the intestines, but will become systematic following translocation across the permeable intestinal mucosa^10^.

The colonisation of nasogastric enteral feeding tubes has been recognised as influencing the neonatal microbiome as well as a possible source of infectious organisms^11^. Such feeding tubes are used for oral feeding of premature babies. The normal feeding regime would be bolus feeding at regular timed intervals of 2–3 h, or continuous feeding using a pump-controlled syringe reservoir of feed. Premature babies will be fed breast milk, or formula through the tube since the normal suckling reaction is not apparent in preterm babies. Previous studies have shown that the tubes quickly become colonised by bacteria and fungi^11,12^. Such tubes are often only semi-transparent and thin, such that bacterial biofilm formation within the tube cannot be visually evaluated.

The neonatal feeding tube flora includes a number of opportunistic bacterial pathogens. Hurrell *et al*.^11^ and Alkeskas *et al*.^13^ showed that 76% (n = 129) of feeding tubes collected from neonatal intensive care units contained biofilms, with up to 10^7^ bacterial colony-forming units (CFU) in a tube. The complex microbial flora included various *Enterobacteriaceae* species (*E. coli* K1, *Enterobacter hormaechei*, *E*. *cancerogenus, Cronobacter sakazakii*, *Serratia marcescens*, and *Klebsiella pneumoniae*), *Pseudomonas fluorescens*, *P*. *luteola*, staphylococci, lactic acid bacteria, as well as fungi such as *Candida albicans*. Many of these organisms such as *E*. *coli* K1, *C. sakazakii*, *E*. *hormaechei*, and staphylococci are recognised as causative agents of severe neonatal infections and can be present in infant formula^14–18^. A quarter of the *E. hormaechei* were resistant to 3rd generation cephalosporins (ceftazidine and cefotaxime), and all *S. marcescens* strains were resistant to amoxicillin and co-amoxiclav. The isolation of *E. coli* K1 strains from feeding tubes is of high clinical significance since it is associated with nosocomial neonatal meningitis. Previously *E. coli* K1 sequence type 95 strains recovered from the feeding tubes of twelve neonates over a three week period were indistinguishable according to pulsed-field gel electrophoresis (PFGE) indicating a unidentified source of exposure, and high risk to neonatal health^13^. Colonization of neonatal feeding tubes by such organisms that can cause highly severe infections of neonates, has not been investigated thoroughly. Additionally, these organisms produce extracellular capsular material associated with biofilm formation, and would entrap other organisms resulting in multi-organism biofilm formation. Hurrell *et al*.^19^ further showed that a single contaminated feed resulted in bacterial biofilm formation within the feeding tube and subsequent contamination of every following feed which would be ingested by the neonate.

Petersen *et al*.^12^ confirmed the rapid colonization of nasogastric tubes which had been in place for 1 day by various *Enterobacteriaceae* and *Staphylococcus aureus*. Eighty-nine percent (n = 94) of such nasogastric tubes contained > 10^3^ CFU/ml bacteria, and of these 55% contained either or both *Enterobacteriaceae* and *S. aureus*. Genotyping of bacterial isolates from nasogastric tubes of individual neonates has revealed the tubes can be colonized by indistinguishable strains irrespective of the feed regime, showing the dissemination of bacteria within neonatal intensive care units (NICU) by personnel and the environment^13^. As the biofilm ages, clumps of bacteria will break off and enter the neonatal stomach, where they will be protected from the low stomach acidity (pH ~ 4.0) due to the bacterial capsular material^11^.

There is evidence for the intestinal colonization by the feeding tube flora, since indistinguishable (according to RAPD and PFGE) bacterial strains have been reported in paired neonatal feeding tubes and faeces^20^. Similarly, nasogastric tube placement was a risk factor for the colonisation by carbapenem-resistance Gram negative bacteria and NDM-producing *A. baumannii* of neonates in intensive care^21^. Both *K. pneumoniae* and *A. baumannii* are members of the ESKAPE group (*Enterococcus faecium*, *Staphylococcus aureus*, *K. pneumoniae*, *A. baumannii*, *P. aeruginosa* and *Enterobacter* species) of bacterial pathogens. These organisms are of increasing clinical concern due to multidrug-resistance and high virulence^22,23^.

Various methods measuring bacterial biofilm formation on surfaces have been reported, but most of these methods involve sample preparation before imaging. Scanning electron microscopy (SEM) of feeding tubes collected from neonates in intensive care show the dense biofilms composed of various bacteria and fungi^11,19,20^. Similar SEM imaging of PVC neonatal enteral feeding tubing with laboratory-generated bacterial biofilm formed by the neonatal pathogen *C. sakazakii* (strain ATCC 12868) revealed clusters of cells on the surface and within a matrix of liquid feed material^18^. However, disadvantages of SEM are the prolonged period of sample preparation and the destructive nature of analysis.

Optical coherence tomography (OCT) is a non-invasive and non-contact method of imaging surface and subsurface microstructures of transparent and semi-transparent materials. OCT was first invented for the *in situ* 3D imaging of the eye^24^ and is being used for an increasing number of clinical and non-clinical applications^25^. The technique is based on the Michelson interferometer using a broadband laser source in the near infrared (NIR) spectral range typically centred at 800 nm and 1300 nm. In general, materials are more transparent in this NIR wavelength range compared with the visible range allowing the returned light to be collected as deep down as 1–2 mm in depth. OCT provides high resolution, spatially resolved images of internal micro-structures of the orders of a few microns or less, comparable to that of histology slide. OCT is most sensitive to the change in refractive index and is therefore very sensitive to layer interfaces. Imaging can be performed *in situ* and in real time. Since this method is non-invasive, it can be used to obtain morphological information of the upper millimetres of tissues. Consequently, it has found clinical applications in ophthalmology, angiography, dermatology, neurology, cancer research, cardiology, as well as organ screening. However, there has only been limited applications to microbiological systems^26–28^.

In this paper, we demonstrate the complementary use of OCT and whole genome sequencing, where the OCT was used for rapid non-invasive visualisation of bacterial biofilm structures and the whole genome sequencing analysis was used to identify and characterise the bacteria inside the feeding tubes of a preterm infant in a NICU.

## Materials and Methods

### Background clinical information

The patient was a low-birth weight (725 g) preterm male neonate with an estimated gestational age (EGA) of 25 weeks and was born by emergency C-section. Two nasogastric feeding tubes were collected during his stay at the NICU. The first feeding tube was collected at 6 weeks of age and had been in place for 10 days, the second feeding tube was collected at age 8 weeks (unknown duration *in situ*). During this period of time the patient had been fed a mixture of fortified breast milk and ready-to-feed formula. On collection, the feeding tubes were divided into two sections, the tip (3 cm) was kept for OCT observation and the adjacent portion (10 cm) for bacteriological analysis. Two faecal samples were collected. The first on the same day as feeding tube 1, and the second 8 days later, and was before the collection of the second feeding tube.

### Bacterial isolation and provisional identification

Bacterial biofilms were sampled as previously described by Hurrell *et al*.^11^. Before sampling, the outside of the feeding tubes were sterilised with ethanol and then sliced open with the biofilm scraped into a sterile tube. Due to ethical approval restriction, the original faecal material could not be stored. Instead, both faecal samples and feeding tube contents were incubated seperately in Brain Heart Infusion (BHI) growth medium overnight at 37 °C. The overnight growth was then mixed with glycerol (20%) and stored frozen (−80 °C) in 1.5 ml aliquots until analysed. Partially thawed samples were streaked on Violet Red Bile Glucose Agar (VRBGA, Fluka-UK) for *Enterobacteriaceae* and incubated under aerobic conditions at 37 °C for 72 h. Samples were also plated onto De Man, Rogosa, Sharpe Agar (MRS, CM0361, Oxoid Thermo Fisher Scientific) for lactic acid bacteria, and Bifidus Selective Media Agar (BSM, 88517 Sigma-Aldrich) for Bifidobacteria. These plates were incubated under anaerobic conditions at 37 °C for 72 h. Representative colonies from each medium were picked according to their morphotype (edge, opacity and texture) and re-streaked for purity on the appropriate medium; VRBGA, BHI, MRS, or BSM, incubated at 37 °C for 24–48 h. Isolates were provisionally identified according to Gram stain morphology, catalase and oxidase reaction, and confirmed using 16 S rDNA sequencing. Draft genomes of selected strains of *Enterococcus faecalis* and *Enterobacter hormaechei* were generated using MiSeq Illumina sequencing as described below.

### Bacterial 16 S rDNA sequence analysis

The aerobic bacterial strains were grown at 37 °C in Tryptic Soy Broth (Oxoid Thermo Fisher Scientific) in a shaking incubator and the anaerobic bacterial strains were grown at 37 °C in Nutrient Broth (Fluka-UK). The DNA extraction was carried out with GenElute™ Bacterial Genomic DNA Kit (Sigma Aldrich) for each strain. Nanodrop 2000 (Oxoid Thermo Scientific, UK) was used to check the concentration and purity of the eluted DNA. The sequencing of the 16 S rDNA loci (528 bp) was performed using the forward primer (TGGAGAGTTTGATCCTGGCTCAG) and reverse primer (TACCGCGGCTGCTG-GCAC). Cycling conditions were initial denaturation at 95 °C for 10 minutes; 30 cycles of denaturation at 95 °C for 30 sec, primer annealing at 62.6 °C for 30 sec, extension at 72 °C for 45 sec; followed by a final extension step of 72 °C for 10 minutes. The sequencing of the 16 S rRNA gene PCR product (approx. 520 bp) was performed for the anaerobic strains using the primers pbl16 (5′-AGAGTTTGATCCTGGCTCAG-3′) and mlb16 (5′-GGCTGCTGGCACGTAGTTAG-3′)^29^. Cycling conditions were an initial denaturation of 94 °C for 5 minutes; 25 cycles of 94 °C for 30 seconds, primer annealing at 48 °C for 30 seconds, extension at 72 °C for 45 seconds and followed by a final extension step of 72 °C for 4 minutes. PCR amplified products were purified using MinElute PCR Purification Kits (Qiagen, UK) by following the manufacturer’s protocol. The DNA concentration was confirmed by using a Nano-Drop 2000 (Thermo Scientific, UK) and sequenced by Source Bioscience Sequencing. Contigs were constructed and compared with the curated Ribosomal Database project (https://rdp.cme.msu.edu/).

### Draft genome sequence generation and analysis

Genomic DNA was extracted from eight *E. faecalis* and six *E. hormaechei* strains using the GenElute™ Bacterial Genomic DNA Kit (Sigma Aldrich) and prepared for whole-genome sequencing using a Nextera XT library preparation kit (Illumina, San Diego, CA). Whole-genome sequencing was performed using the Illumina Miseq reagent kit v3 (250-bp paired-end reads). The reads were assembled *de novo* using Spades^30^. Genome annotation was performed using Prokka (http://www.vicbioinformatics.com/software.prokka.shtml).

Speciation of the strains were confirmed by core-genome alignment using parsnp and the phylogenetic trees visualised with Gingr, both programs belong to the Harvest package^31^.

Single nucleotide polymorphisms (SNPs) were identified using the program CSI Phylogeny 1.4 available on the CGE website (http://www.genomicepidemiology.org/).

Virulence traits and antibiotic resistance genes were identified using NCBI BLASTn, VirulenceFinder 1.5, ResFinder 2.1 software (both available on the CGE website) and Artemis browsing using reference virulence genes obtained on the Virulence Factors database website (www.mgc.ac.cn/VF).

### Imaging with Optical Coherence Tomography (OCT)

The optical fibre-based ultra-high resolution (UHR) spectral domain OCT system was developed in-house for imaging sub-surface structure at an axial resolution of the order of 1 micron^32^. To achieve UHR in the depth direction, a supercontinuum source (NKT SuperK Versa) was used. A fibre based Michelson interferometer was used and the interference signal was recorded by the spectrometer which was designed with a 1200 l/mm transmission grating and a E2V AViiVA 4010 EM4 linear CCD array. The data recorded by the spectrometer was then resampled into equal k-spacing and fast Fourier transformed to obtain the A-scan depth profile. A pair of scanning mirrors in the probe head allows the collect of a volume.

The UHR OCT has a central operating wavelength of 810 nm with depth profile acquisition speed of 37 kHz which gives a maximum 2D image (500 lines) frame rate of 74 fps. The probe head has a working distance of about 4 cm. The system has an axial resolution of ~1.2 μm (in polymer) over a depth range of 1.6 mm^32^. The transverse resolution is 7 μm given by the objective lens used. The sensitivity decrease with depth is within 2 dB over a 1.2 mm depth range. The high resolution and sensitivity of the system makes it convenient to image thin and turbid layers such as biofilm inside a semi-transparent tube (it is opaque in the visible but semi-transparent in the NIR) with good contrast. In order to maintain the axial resolution, dispersion due to the tube wall was compensated for by adjusting the length of the dispersion compensation glass.

The proximal 3 cm end of each feeding tube (including the holes) was washed in ethanol and air-dried at room temperature. In addition, a negative bacterial control tube was prepared by flushing 2 ml of ready-to-feed infant formula through a sterile feeding tube, and incubating at 37 °C overnight and air-dried before being imaged by the OCT. The tubes were then scanned using the OCT at 5 mm intervals along the length of the tubes, which were rotated around the centre of the tube axis every 90° for a maximum view of the inside wall of the tube.

The datasets generated and/or analysed during the current study are available from the corresponding author on reasonable request. The parents gave their informed consent for material/information from the babies to be used. The work reported with neonatal information is in compliance with the ethical approval of the regional health authority.

### Ethics

Ethical approval was given by NRES Committee East Midlands. This permitted the collection of feeding tubes, faeces and metadata, but did not permit the storage of human tissue which by definition included faeces.

## Results

### OCT analysis

**Figure 1:**
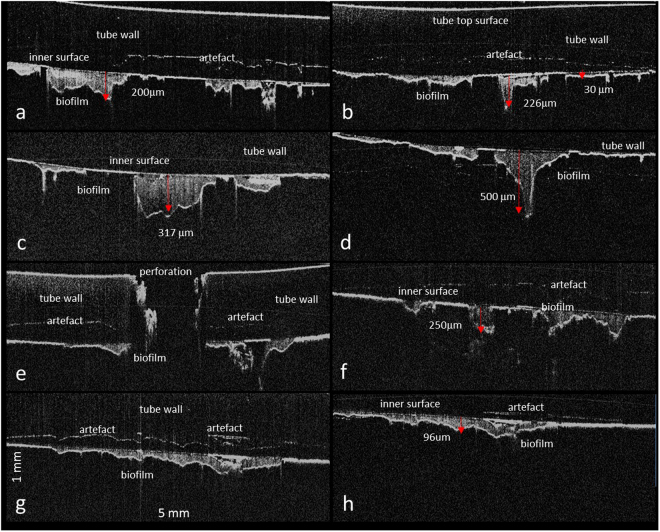
OCT virtual cross-section images showing the used feeding tube taken from the antenatal care unit. The images are 5 mm across and 1 mm in the depth direction and they show the biofilm accumulation taken at various positions of the feeding tube with the thickness of the biofilm marked at selected points.

**Figure 2:**
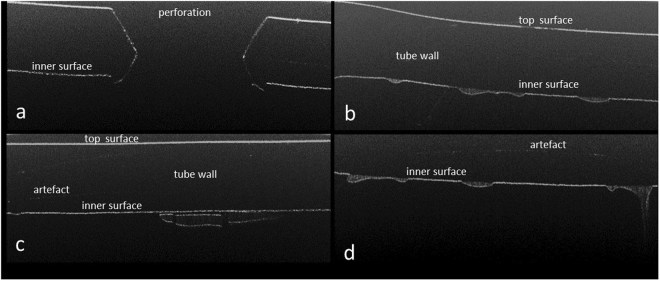
OCT virtual cross-section images of the control sample of feeding tubes incubated overnight with sterile ready-to-feed formula and then dried. These images were taken at various positions on the feeding tubes and are 5 mm across and 1 mm in depth.

Feeding tubes from the NICU contained irregular areas of dense heterogeneously distributed material inside the feeding tubes (Fig. 1). These were found close to the holes at the terminated end of the tubes. OCT images were obtained for 5 mm along the length of the tube and 1.6 mm in depth portions of the feeding tubes. This data set was then used to quantify the thickness of the dense areas attached to the inner wall of the feeding tube. They varied in thickness between 20 and 500 µm, as well as in length, between 438 µm and 3.5 mm. Many separate biofilm areas could be observed in close proximity to each other. The artefacts marked in Fig. 1 are due to the interference between the bright reflective top surface of the tube and the bright interfaces at the inner wall of the tubes. These artefacts can be distinguished from the real interfaces by moving the sample relative to the probe. The position of the artefacts will not change as the sample moves relative to the probe, but the real image will move vertically. In contrast, the *in vitro* generated negative bacterial control had mostly low scattering, homogeneous thinner layers of material attached to the inner surface of the feeding tube (Fig. 2). The appearance of the OCT images of the neonatal feeding tubes and their negative control samples were distinctively different and could be easily distinguished.

### Bacteriological analysis

Bacteria were recovered on the three types of agar plates for *Enterobacteriaceae* and lactic acid bacteria. Three of each colony morphotype was selected for identification as according to Gram strain reaction, catalase and oxidase test and 16 S rDNA sequence analysis. Selected strains were then subject to whole genome sequencing and their identification confirmed using core genome phylogeny (data not shown). The genomes were then uploaded to the Center for Genomic Epidemiology database to predict antimicrobial resistance, virulence characteristics and sequence type.

### Bacterial genomic analysis of E. faecalis strains

The genomic profiling of *E. faecalis* revealed that three isolates (2321, 2325, 2328) belonged to ST191 and five isolates (2322, 2323, 2324, 2326, 2329) to ST211. *E. faecalis* ST191 strains had been isolated from feeding tube 2 only, whereas the ST211 isolates had been recovered from feeding tube 1, as well as faecal samples 1 and 2 (collected 8 days apart). All genomes encoded for macrolide resistance (*lsaA*) and tetracycline resistance (*tetM*). The two sequence types differed in their capsular type, according to the presence of *cps* genes; ST191 only encoded for *cpsAB*, whereas ST211 encoded for the entire *cps* gene cluster except for *cpsF*. The genomic analysis also revealed the presence of various adherence and biofilm-related traits; *ace, ebp* pili, *efaA, bopD*, as well as exoenzymes (gelatinase, hyaluronidase and *sprE*). These traits did not differ between sequence types.

**Figure 3:**
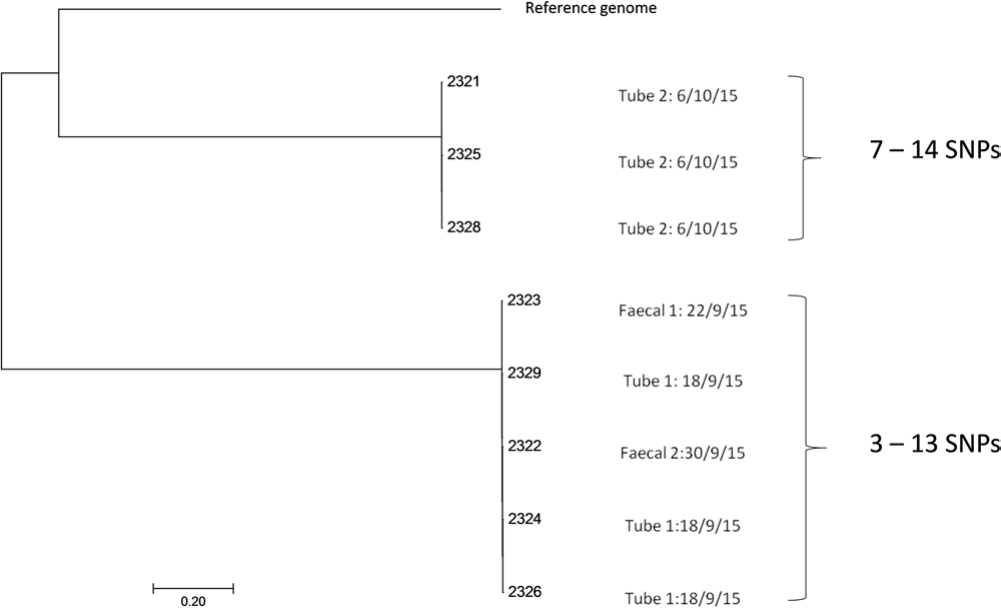
Single nucleotide polymorphism analysis tree of *Enterococcus faecalis* isolates.

SNP difference determination confirmed that the *E. faecalis* isolates formed two distinct clusters, as according to their sequence type (Fig. 3). The five *E. faecalis* ST211 isolates had 3–13 SNPs difference. These strains were isolated 12 days apart in total, 4 days between the first feeding tube and the first faecal sample, and 8 days between the first feeding tube and the second faecal sample. The three *E. faecalis* ST191 strains had 7–14 SNPs difference between them. These had been isolated from the same feeding tube (2), about 2 weeks after the ST211 isolates. There were *ca*.12000 SNPs difference between the two clusters of ST211 and ST191 strains (Fig. 3).

### Bacterial genomic analysis of E. hormaechei strains

Six *E. hormaechei* strains were subject to whole genome sequencing and closely clustered with *E. hormaechei* subsp. *steigerwaltii* using core genome phylogeny (data not shown). Genome analysis using the Center for Genomic Epidemiology database showed they all belonged to ST106, and encoded for *bla*ACT-15 and *fosA*.

**Figure 4:**
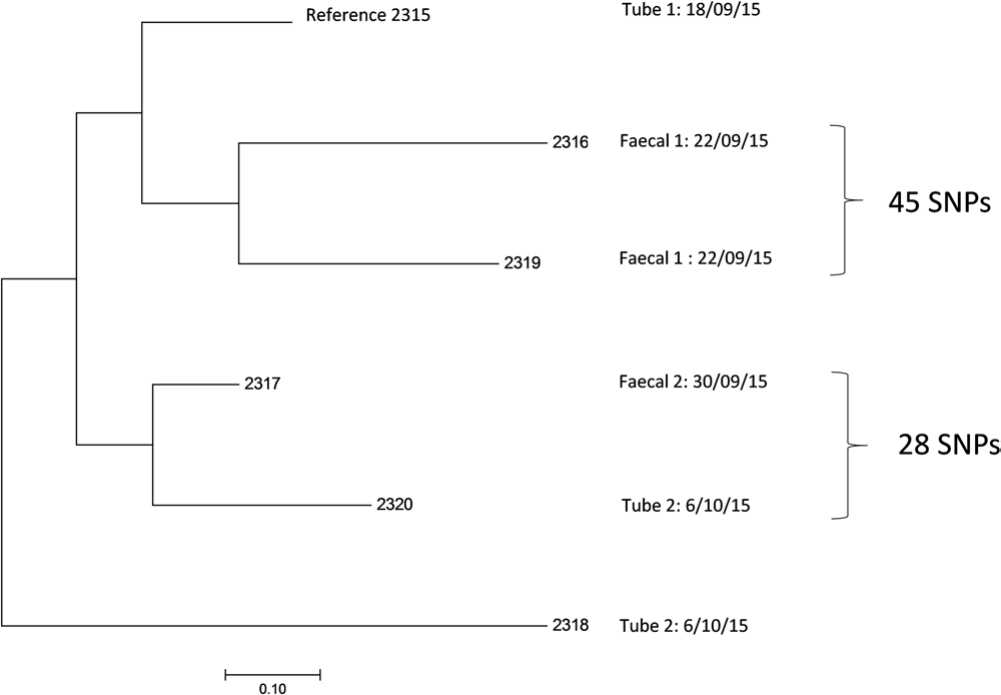
Single nucleotide polymorphism analysis tree of *Enterobacter hormaechei* isolates.

The six *E. hormaechei* strains differed from each other by 28–70 SNPs and had been obtained over a 1 month period (Fig. 4). Two *E. hormaechei* isolates (2316 & 2319) which had been obtained from the same faecal sample (1), differed by 45 SNPs. The two isolates (2320 & 2318) from feeding tube 2 had 59 SNPs difference. It was notable that the two strains with the lowest SNPs difference (28) were from two separate samples; 2317 (faecal sample 2) and 2320 (feeding tube 2). These two samples had been obtained 6 days apart.

## Discussion

The initial colonization of the infant gut begins at birth when the infant is exposed to the mother’s vaginal and faecal microbiota. It will then alter according to the feeding regime (breast milk and prepared formula), as well as exposure to the carer’s skin flora and their environment^33^. The neonatal flora has an importance in the formation of the gut barrier and immune system development. Contributors to the ingested flora are generally regarded as the mother’s milk and microbiota, but the intermediate flora of the feeding tube where bacterial can flourish has not been studied in detail. While the microbiological safety of infant formula feeding has been re-evaluated in order to reduce the ingestion of potentially pathogenic bacteria such as *Cronobacter* spp.^34^, the accumulation of bacteria inside feeding tubes has not received attention. Such tubes become heavily colonized by bacteria while *in situ*, and can act as loci for bacterial transmission to the neonate following ingestion of bacterial clumps from biofilm formation inside the tube lumen^8,11^.

This study is the first to show that bacterial biofilms in used neonatal feeding tubes can successfully be visualized non-destructively using OCT. The bacterial biofilms were heterogeneously distributed along the inside wall of the tubes (Fig. 1). Their appearance was clearly distinguishable from that of milk residues as shown in the negative control (Fig. 2). The depth of the biofilms was quantifiable and was as much as 500 µm. Given the normal size of bacteria (1 × 2 µm), considerable numbers of bacterial cells can be present in these layers. The variation in shading of the bacterial biofilm indicates that they are heterogeneous in their composition and may reflect the fluid channels, as previously proposed within bacterial biofilms using destructive methods of analysis^35,36^. Future studies could include the comparative quantification of bacterial biofilms between feeding tubes and catheters of different materials (including antimicrobial composites), feeding regimes and in-place duration.

The presence of both Gram-positive and Gram-negative bacteria inside neonatal feeding tubes has previously been reported^7,8,11,12,20^. However, the contribution of bacterial colonization in the feeding tubes to the colonization of the infant intestines has not been studied in detail. In order to demonstrate that the bacterial biofilms seen in OCT images were composed of viable organisms, the layers were sampled for microbiological analysis. Furthermore, the possible influence of the feeding tube flora on the neonatal intestinal flora was investigated by sampling faecal material from the same neonate, followed by identification and whole genome analysis of isolates. The neonate was in intensive care due to their premature birth (EGA 25 weeks) and the samples were collected at weeks 6 & 8 after birth.

In order to specifically investigate the dual colonisation of the feeding tubes and intestines, only strains of *Enterococcus faecalis* and *Enterobacter hormaechei* were collected from the selective media for detailed analysis. *E. faecalis* was chosen as it is a common bacterium in the human intestines (10^4^-10^8^ cfu/g) and an early coloniser of the infant intestinal tract^37^. They are also opportunistic pathogens and encode for intrinsic and acquired antimicrobial resistance. Consequently, they are a major cause of antibiotic-resistant infections and are amongst the most common nosocomial pathogens infecting the bloodstream, surgical sites and urinary tract^38,39^. *E. hormaechei* was also chosen as it is a bacterial pathogen of increasing concern due to reports of neonatal infections and it encodes for antibiotic resistance. Also, it has been misidentified in reported neonatal cases of *C. sakazakii* infections^10,30,40^. Both organisms are also members of the ESKAPE group^22,23^.

Eight *E. faecalis* isolates from the feeding tubes and faecal samples were identified and subjected to whole genome sequencing (Fig. 3). Genomic analysis assigned three strains as ST191 and the remaining five strains as ST211. SNP analysis clustered the three ST191 isolates (2321, 2325 & 2328) from the same tube (tube 2) together. These isolates had only 7–14 SNPs difference between them and were therefore considered as multiple isolations of the same ST191 strain. The remaining 5 isolates (ST211) clustered together. These were from both feeding tubes and faecal material collected on different weeks. These five isolates had only 3–13 SNPs difference between them and were *ca*. 12000 SNPs different from the first cluster (ST191 strains). Given the small number of SNP differences, they were regarded as nearly indistinguishable isolates of the same *E. faecalis* ST211 strain. This strain had persisted in the feeding tube, and had also colonised the infant’s intestines. Since the feeding tube had been changed between the two sampling points, the organism could either have colonised the throat or had re-inoculated the stomach due to reflux^11^. The common practice of checking the location of the feeding tube by determining the pH of the feeding tube contents (acid indicating stomach as opposed to lung) may be inadvertently contaminating the newly placed tube with bacteria from the stomach.

Genome analysis of the *E. faecalis* strains revealed a number of encoded clinically-relevant characteristics. All genomes encoded for macrolide resistance (*lsaA*) and tetracycline resistance (*tetM*) and therefore potential resistance to antibiotic treatment following neonatal infection. All strains encoded for adherence traits; *ace* and Ebp pili (*ebpABC, srt, ecbA*) which could aid their attachment to the host intestinal cells. The multiple ST211 strains also encoded for the capsulation genes *cpsA-G*. Their presence designates the strains as having the CPS 2 capsule type, whereas the ST191 strains only encoded for *cpsAB* which corresponds to CPS 1 capsule type^40,41^. Functional capsular polysaccharide is expressed by the CPS 2 type, but not the CPS 1 type. Capsulation is relevant for attachment and biofilm-formation in the feeding tube and subsequent increased ingestion. Thus enabling host immune system evasion and exposure to the neonate. In addition, it is plausible that the production of lactic acid following the fermentation of sugar by the enterococci causes the precipitation of casein in the breast milk or formula in feeding tubes. The coagulated protein could then cause bacterial entrapment and adhesion to the feeding tube wall, enhancing bacterial biofilm formation. Given the first tube had been in place for 10 days, there was substantial opportunity for bacterial colonisation in the feeding tube and subsequent ingestion by the neonate of bacterial clumps from the biofilm.

A total of 6 *E. hormaechei* isolates were recovered from feeding tubes and faecal samples. All strains were ST106, encoded for the same antibiotic resistance traits (*bla*ACT-15 and *fosA*), and had only 70 SNPs difference between them. Given the relatively small number of SNPs differences they could be considered as closely related strains of the same sequence type which have diverged during colonisation of the neonate and feeding tubes. Their presence confirms the apparent persistent colonisation of neonatal feeding tubes and intestines by members of the early flora as shown with *E. faecalis*. Since *E. hormaechei* and *E. faecalis* are opportunistic pathogens, and hence a potential risk to infant health, such persistence warrants further investigation with larger cohort studies to determine the significance of these findings.

This study has also shown the quantitative application of OCT to bacterial biofilm formation on *in vivo* feeding tubes from a neonate in intensive care. Being able to quantify bacterial biofilm formation using OCT *in situ* on the feeding tubes without contact and without the need for sample preparation will enable future studies determining the efficacy of antimicrobial treatments. The measurement of which, currently suffers from the destructive nature of analysis, restricting re-analysis and representative analysis of heterogeneous biofilm formation. In addition, OCT is demonstrated to have the potential for rapid, *in situ* examination of the feeding tubes for biofilm growth in the feeding tubes.
